# Mobile Phone Detection of Semantic Location and Its Relationship to Depression and Anxiety

**DOI:** 10.2196/mhealth.7297

**Published:** 2017-08-10

**Authors:** Sohrab Saeb, Emily G Lattie, Konrad P Kording, David C Mohr

**Affiliations:** ^1^ Center for Behavioral Intervention Technologies (CBITs) Department of Preventive Medicine Northwestern University Chicago, IL United States; ^2^ Rehabilitation Institute of Chicago Department of Physical Medicine and Rehabilitation Northwestern University Chicago, IL United States

**Keywords:** semantic location, geographic positioning systems, mobile phone, classification, decision tree ensembles, extreme gradient boosting, depression, anxiety

## Abstract

**Background:**

Is someone at home, at their friend’s place, at a restaurant, or enjoying the outdoors? Knowing the semantic location of an individual matters for delivering medical interventions, recommendations, and other context-aware services. This knowledge is particularly useful in mental health care for monitoring relevant behavioral indicators to improve treatment delivery. Local search-and-discovery services such as Foursquare can be used to detect semantic locations based on the global positioning system (GPS) coordinates, but GPS alone is often inaccurate. Mobile phones can also sense other signals (such as movement, light, and sound), and the use of these signals promises to lead to a better estimation of an individual’s semantic location.

**Objective:**

We aimed to examine the ability of mobile phone sensors to estimate semantic locations, and to evaluate the relationship between semantic location visit patterns and depression and anxiety.

**Methods:**

A total of 208 participants across the United States were asked to log the type of locations they visited daily, using their mobile phones for a period of 6 weeks, while their phone sensor data was recorded. Using the sensor data and Foursquare queries based on GPS coordinates, we trained models to predict these logged locations, and evaluated their prediction accuracy on participants that models had not seen during training. We also evaluated the relationship between the amount of time spent in each semantic location and depression and anxiety assessed at baseline, in the middle, and at the end of the study.

**Results:**

While Foursquare queries detected true semantic locations with an average area under the curve (AUC) of 0.62, using phone sensor data alone increased the AUC to 0.84. When we used Foursquare and sensor data together, the AUC further increased to 0.88. We found some significant relationships between the time spent in certain locations and depression and anxiety, although these relationships were not consistent.

**Conclusions:**

The accuracy of location services such as Foursquare can significantly benefit from using phone sensor data. However, our results suggest that the nature of the places people visit explains only a small part of the variation in their anxiety and depression symptoms.

## Introduction

Passive and unobtrusive detection of the physical location of individuals has been made possible over the years by embedding global positioning system (GPS) systems into commonly used devices, such as mobile phones. Physical location alone is usually not very useful for understanding human activity, or the motivations that underlie that activity. In contrast to physical location, semantic location carries additional information about the meaning of the location [1]. For example, semantic location might tell us if a location is a home, place of work, dining establishment, or place of worship, thereby infusing the geographic location with human relevance.

A growing number of papers have shown that a variety of location features, measured by GPS, can detect mental health problems such as depression [2-6], bipolar disorder [7], and social anxiety [8]. It is unclear at this point why these GPS location features may be related to depression or anxiety. It may be that the nature of the places and the meaning inherent in different locations affect how we feel. Previous research has shown that there is a relationship between mood and certain activities, such as religious practice [9], participating in social activity [10], and spending excess sedentary time at home [11]. Improving the ability to detect locations affiliated with these activities could offer not just a greater understanding of the behavioral and environmental contributors to depression and anxiety, but also unique methods for prompting just-in-time adaptive interventions (JITAIs) using mobile technologies. This approach could add value beyond that gathered using other JITAI triggers (eg, self-reported difficulties, GPS location, and electro-cardiogram signals), and may enable us to determine if a person with a history of depression is relapsing, or if a person is about to have a panic attack [12].

Local search-and-discovery services, such as Foursquare, can estimate semantic locations based on GPS coordinates and the data they have globally collected from populated areas in the world. When these services are embedded in a mobile app using an application programming interface (API), they can passively provide location-specific information for the locations that users visit. Since its launch in 2009, Foursquare has been used in research applications to accomplish diverse tasks, ranging from the analysis of individuals’ food and drink habits across cultures [13] to the examination of the popularity of venues and identifying factors contributing to venue popularity [14]. Foursquare has tapped into a new model of location-based advertising such that users can be notified of businesses in their immediate vicinity, and can receive benefits such as discounts and coupons for “checking in” to these businesses.

However, asking search-and-discovery services such as Foursquare about semantic locations, based on a given GPS coordinates, has limitations. First, GPS can be inaccurate, and particularly in denser urban environments, variability in GPS may lead to the detection of false locations. For example, one might be at a restaurant within a shopping mall, and the search-and-discovery service may classify the person as at a shop rather than a restaurant. Second, although these services can detect “residential” locations, they cannot distinguish a person’s home from another home they are visiting. These limitations prevent such services from being a reliable source of information, especially for behavioral sensing and intervention, where it is crucial to know exactly when a person is at home, work, a friend’s home, or other locations.

In addition to GPS, mobile phones can sense many more variables in the environment, such as light, sound, and Wi-Fi signals. Using a mobile phone, we can also determine what type of physical activity an individual is performing, how much time they spend in a location, and how they interact with their phones. Semantic locations may have distinct signatures, such as the length of time a person spends at a location, time of the day and day of the week that they visit, type of activities that they perform, and the sound and light conditions in the environment. These features may help us to determine if the place is home, a grocery store, place of worship, or a library. As an obvious example, a place that a person spends time over night is most likely home, and a bright place visited during the day, with intermittent walks and stops, is likely a store. Therefore, detection of semantic locations using mobile phone sensors seems feasible.

The aim of this paper was first to develop methods for improving mobile phone-based detection of semantic locations by incorporating sensors beyond the simple GPS. We developed methods for detecting semantic locations, and compared their accuracy to that of Foursquare. While improving semantic location detection is worthwhile and could further serve clinical and consumer-driven purposes, our second aim was to explore the relationship between semantic location detection and depression and anxiety. We specifically investigated the relationship between semantic location visits and the severity of depression and anxiety symptoms, as well as the differences between individuals with and without those symptoms.

## Methods

### Participant Recruitment

We recruited participants between October 28, 2015 and February 12, 2016. The recruitment was done in collaboration with Focus Pointe Global (FPG), a company that specializes in market and scientific research strategies and participant recruitment and retention [15]. FPG maintains a panel of 1.5 million potential participants from the general population. For our study, FPG sent out emails to potential participants with links to the screener questionnaire. Additionally, FPG used phone calls to contact potential participants from their in-house registries.

Interested individuals from the general population of the United States contacted FPG and were screened for eligibility using a brief questionnaire. Individuals were eligible for our study if they were at least 18 years old, able to read and understand English, owned a mobile phone with Android 4.4 through 5.1, and had access to Wi-Fi for at least one 3-hour period per day. We excluded individuals who indicated on self-report that they were diagnosed with any psychotic disorders, were unable to walk more than half a mile (4 city blocks), or had positive screens for alcohol abuse (Alcohol Use Disorders Identification Test [16] score >16), drug abuse (Drug Abuse Screening Test-10 [17] score >6), suicidal ideation (Beck Depression Inventory-II [18] item 9 rating >2), or bipolar disorder (Mood Disorder Questionnaire [19] question 1 score 7, an endorsement of question 2, and a response of 2 or 3 for question 3). We also excluded individuals who shared their phone with others.

Depressive symptoms were measured using the Patient Health Questionnaire, 9-item (PHQ-9) [20]. On the PHQ-9, participants are prompted to indicate how frequently they have experienced specific symptoms over the past two weeks, such as “feeling down, depressed, or hopeless” and “feeling tired or having little energy”. Participants respond on a four-point Likert-type scale, ranging from 0 indicating “not at all” to 4 indicating “nearly every day.” PHQ-9 scores range between 0-27. We also used the cut-off point of 10 to divide participants into those who screened positive for depression (PHQ-9 >10; termed *depressed* in this paper) and those screened negative (PHQ-9 <10; termed *nondepressed*). This cut-off point has been shown to maximize the sum of sensitivity and specificity for depression diagnosis [20].

For anxiety assessment, we used the Generalized Anxiety Disorder, 7-item (GAD-7) [21]. The GAD-7 is structured similarly to the PHQ-9, and participants are prompted to indicate how frequently they have experienced symptoms such as, “feeling nervous, anxious, or on edge” and, “being so restless that it’s hard to sit still” over the past two weeks on the same four-point Likert-type scale. GAD-7 scores range between 0-21. We used the cut-off point of 10 to separate those participants who screened positive for GAD (GAD-7 >10; termed *anxious* in this paper) from those who screened negative (GAD-7 <10; termed *nonanxious*). At this cut-off point, the sum of sensitivity and specificity is maximized [21].

We wanted to have a wide range of depression and anxiety symptoms in our sample, and therefore we selected roughly equal numbers of participants in four groups, based on their screening assessments: depressed and anxious, depressed and nonanxious, nondepressed and anxious, and nondepressed and nonanxious. In addition to assessment at baseline, we also assessed each participant’s depression and anxiety at week 3 and week 6.

### Participant Enrollment

Eligible participants were consented using procedures approved by the Northwestern University Institutional Review Board. Consenting was done using a website: participants were directed to a webpage that contained information about the study procedures, benefits, and potential risks. Specifically, participants were informed about the sensor data that were going to be collected from their mobile phones, the types of questions that would be asked throughout the study, and the procedures undertaken to protect their private information. After digitally signing the consent form, participants were enrolled in our study.

Each participant was enrolled for a period of 6 weeks. First, a study identification (ID) number was assigned to the participant by FPG. Participants were then asked to complete an online questionnaire regarding their demographic information, which consisted of their age, gender, race, and ethnicity, along with their US state of residence, and information about various aspect of their lives that could impact movement patterns (eg, health difficulties, number of jobs, and job locations). Finally, participants downloaded two apps: *Purple Robot* [22], which collected sensor data from their phones; and *EMA app*, which asked them questions about the places they visited. Participants were compensated between US $25 and $270.40 depending on how long they stayed in the study and how many of the daily questionnaires they answered.

### Mobile Phone Data Collection

After participants were enrolled, we started collecting two categories of data from their mobile phones: (1) sensor data, which contained data from the physical sensors as well as software services such as phone and short message service (SMS) communications; and (2) ecological momentary assessment (EMA) data, which consisted of daily questions that showed up on participants’ phones asking them about the locations they visited throughout the day.

The phone sensor data were captured using the *Purple Robot* [22] app. Purple Robot is a multi-purpose, open-source Android app that we have developed for passive collection of mobile phone sensor data [3]. This app gathers data from the sensors and services available on the phone, including light, sound, GPS, accelerometer, phone and SMS communications, screen, and Wi-Fi. The app initially stores sensor data on the device, and then transmits them as network connectivity becomes available. This strategy allows us to collect data in a variety of wireless connectivity scenarios with the confidence that intermittent network access does not affect the nature, quality, or quantity of the collected data.

For the collection of EMA data, we used a second Android app, *EMA app*, which asked participants questions about the locations they visited throughout the day. The app was specifically developed for this study. Each evening, the app analyzed the GPS data collected over the previous 24 hours. The EMA app first clustered the GPS data using an adaptive *k*-means clustering method [3], considering a maximum radius of 100 meters for each cluster, and then removed the clusters that the user visited for a duration of less than 10 minutes. This second step removed clusters that were not actual locations, but were generated because the user was moving slowly (eg, they were stuck in the traffic). After detecting the visited locations, the EMA app provided the participant with a map identifying each location, the time they were at the location, and asked the following questions: “What is the name of this place?” and, “What kind of place is this?”

#### What is the Name of This Place?

A list of likely location names was provided to the user to choose from. This list was obtained from the Foursquare location API. The participant could also enter their own location name if it was not provided.

#### What Kind of Place is This?

This list was adapted from Foursquare venue categories, and included Arts & Entertainment, Food, Nightlife Spot, Outdoors & Recreation, Professional or Medical Office, Spiritual, Shop or Store, Travel or Transport, and Home. In addition, we added Work, Another's Home, and Other. If the participant answered Other, they were asked to enter the location type. The EMA app saved the cluster center corresponding to each detected location, the visit times, and the participant’s answers to the questions regarding that location. Table 1 lists the location categories we used in the EMA app, and how they matched Foursquare’s high-level location categories.

**Table 1:** Location category labels reported by our study participants (left) and their corresponding high-level Foursquare location categories.

EMA app Location Category	Foursquare Location Category
Nightlife Spot (Bar, Club)	Nightlife Spot
Outdoors & Recreation	Outdoors & Recreation
Arts & Entertainment (Theater, Music Venue, Etc.)	Arts & Entertainment
Professional or Medical Office	Professional & Other Places
Food (Restaurant, Cafe)	Food
Home	Residence
Shop or Store	Shop & Service
Travel or Transport (Airport, Bus Stop, Train Station, Etc.)	Travel & Transport
Work	-
Another’s Home	-

Purple Robot and EMA app anonymized any sensitive information before storage and transmission. Specifically, the apps used an MD5 hashing algorithm [23] to anonymize the study participant identifiers. Once the data was anonymized, it was transmitted to the data collection server, and the local copy was deleted from the device. The data residing on the server could be linked with other information gathered during the study only if the unique identifiers used by the participants and the study-specific keys used to encrypt the data were known.

### Foursquare Evaluation

We wanted to assess how well Foursquare could predict the type of locations that users reported daily. To do so, we used the Foursquare wrapper library [24] in Python, and queried the type of location for each location that participants visited. These queries used 4 parameters: latitude, longitude, database version date, and limit. For latitude and longitude, we used the GPS coordinates of the visited location that was saved by the EMA app. For the database version date, we used the current date at the time of the query, which was 2016/8/10, so that we had the latest version of the data. The limit parameter indicated the number of guesses, for which we used 1, so that it returned the best match. We performed these queries for each of the visited locations recorded by EMA app.

Foursquare’s response to our queries was in JavaScript object notation (JSON) format [25], and contained the place ID, name, contact information, address, distance from the queried coordinates, and the location category. From this information, we only saved location category and distance.

The location category returned by the Foursquare website was too specific, being as detailed as “Cambodian Restaurant” or “College Math Building”. Since we did not need this level of detail in our study, we used Foursquare’s Category Hierarchy [26] to translate these low-level categories into high-level ones. This category hierarchy can be obtained in JSON format using the HTTPS query detailed in Textbox 1. The response contains the whole category hierarchy.

**HTTPS query for category hierarchy.**

https://api.foursquare.com/v2/venues/categories?oauth_token=<TOKEN>&v=<VERSION>

Where TOKEN can be obtained from Foursquare’s developers’ website, and VERSION is the database version date in YYYYMMDD format

After querying the Foursquare category for each location cluster, we compared it to the category reported by the participant, and calculated the accuracy (see section: Classifier Evaluation). We skipped locations reported as Work, Another’s Home, or Spiritual for this comparison, since these did not exist in Foursquare categories. The calculated accuracy gave us the performance of Foursquare in predicting semantic locations.

### Detecting Semantic Location from Phone Sensor Data

#### Sensor Features

To classify semantic locations from phone sensor data, we first calculated their features. These features were extracted from all sensor data that were gathered during a visit to a location. In this way, for every location visit, we obtained one feature vector. This vector consisted of 45 features, which will be described in the following sections.

##### Light Features

Light features were calculated from light intensity, in *lux*, sampled by the light sensor at 10 Hz. This sampling frequency could vary from device to device, so light features were designed such that they did not depend on the sampling frequency. These features consisted of basic statistics including mean, variance, skewness, and kurtosis. In addition, we calculated the percentage of time the light sensor output was zero, and the number of times that it crossed its mean value in 1 second.

##### Sound Features

**Figure 1:**
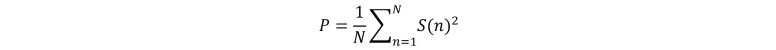
Sound power calculation; where S(n) is the sound amplitude (dB) at sample n, and N is the total number of samples.

Sound features captured different aspects of the sound in the environment. Specifically, we sampled the audio using the phone’s microphone every 5 minutes, each time for 15 seconds. From each 15 second audio recording, we extracted the power and the dominant frequency. Power was calculated as described in Figure 1.

To calculate the dominant frequency, we obtained the amplitude of the fast Fourier transform of the audio signal, and found the frequency that maximized the amplitude.

##### Screen Features

We used screen activity to measure the amount of participants’ interaction with their phones. We calculated the number of times the screen state transitioned from *OFF* to *ON*, as well as the average and the standard deviation (SD) of the duration that the screen was *ON* each time.

##### Activity Features

We used the physical activity states provided by the Android Activity Recognition API. We sampled this API every 10 seconds. The Physical Activity API uses the accelerometer sensor to detect the following physical activities: *Still*, *Walking*, *Running*, *Tilting*, *On Bike*, *In Vehicle*, *Unknown*. We calculated the percentage of time that the participant was in *Still*, *Tilting*, *Walking*, and *Unknown* states. In addition, we calculated the percentage of transitions for a number of state transitions that we expected to be informative about the type of location the participant was visiting. These transitions included *Still* to *Walking*, *Still* to *Tilting*, *Still* to *Unknown*, and *Walking* to *Unknown*.

##### Communication Features

Communication features consisted of the total number of incoming, outgoing, and missed phone calls. In addition, we derived the number of incoming and outgoing SMS text messages.

##### GPS Features

**Figure 2:**
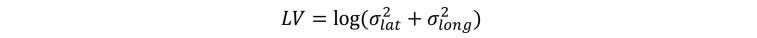
Location variance feature; calculated as the logarithm of the sum of variances in latitude and longitude values.

These features were calculated from the latitude and longitude values provided by the GPS sensor, sampled every 5 minutes. GPS features included average latitude, average longitude, and *location variance* defined as the equation in Figure 2.

In addition to these features, by filtering out the data points that were outside the 50-meter radius of a location’s average latitude and longitude during a visit, we approximated the *visit frequency* to that location, and the *mean time interval* between the visits.

##### Wi-Fi Features

We sampled the current access point’s media access control address and the number of available Wi-Fi networks every 5 minutes. We only used the number of Wi-Fi networks as a feature.

##### Time Features

We calculated the visit duration, the *timespan* of the visit, the visit mid-time in hour, and the day of the week at the start and the end time of the visit. Visit duration was defined as the total time a participant spent at a location on a given day, while visit timespan was the time from when they entered that location first on a given day to the time they left it on the same day.

##### Weather Features

We obtained the weather conditions at the location and time of visits. For this data, we used the Weather Underground service [27]. For each detected location, we queried Weather Underground for the history of weather data in that location, which returned those data for the past year from the date of query. The responses were in JSON format, with each entry corresponding to one weather report. We searched for the report that was closest to the time the user visited that location, and used the temperature, dew point, and weather condition as features.

#### Classifier Architecture

We wanted to see how successfully we could detect semantic locations, reported by the participants, using the sensor features that were passively collected from their mobile phones. For this classification problem, we used ensembles of decision trees with the gradient boosting optimization method [28], also known as extreme gradient boost (XGBoost). These classifiers have been shown to outperform other classification methods in high-dimensional machine learning problems [29]. In this study, we particularly chose XGBoost because these classifiers perform well when the dimensionality of the data relative to the number of samples is large [30], and that they can deal with missing values.

**Figure 3:**
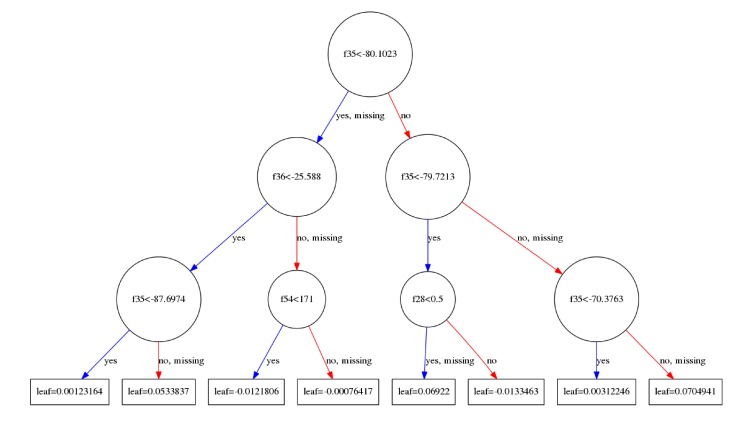
An example of a single decision tree in the ensemble of decision trees. Each circle is a tree node, where a decision is made by comparing a feature value fxx to a threshold. For a given feature vector, depending on which path is taken, a single prediction score is generated, shown in the boxes. Note that one of the outgoing branches of each node is also dedicated to the situation where the data is missing.

A decision tree, shown in Figure 3, determines the class of a feature vector by making sequential, individual decisions on the elements of that vector. Each decision is made at a *node*, where the value of one feature is compared to a threshold value. The node has two outgoing branches that reach next-level nodes. Depending on whether the feature value is larger or smaller than the threshold, one of the branches is chosen. One branch is also designated to the condition where the feature value is missing.

**Figure 4:**
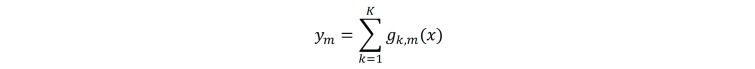
Aggregation of prediction scores made by individual trees; where gk,m represents the decision tree k in class m, that maps a feature vector x to a prediction score gk,m(x), and ym is the ensemble prediction score for class m. K is the total number of trees for each class.

Each decision tree in the ensemble is assigned to one class, and provides a *prediction score* at its leaf node (Figure 3, boxes) for the class it belongs to. The ensemble’s prediction score for each class is calculated by summing over the prediction scores of all trees in that class, as detailed in Figure 4.

**Figure 5:**
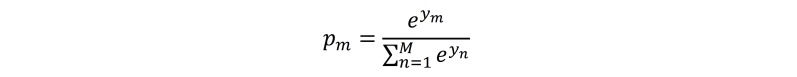
Class probability calculation; where pm represents the probability for class m, ym is the ensemble prediction score for class m, and M is the number of classes.

The final class probabilities are calculated as a softmax function of the predictions scores using the equation shown in Figure 5.

Therefore, for each given feature vector, the ensemble provides a probability distribution over the classes.

#### Classifier Training

**Figure 6:**
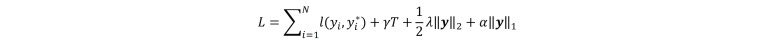
General form of the cost function; where l(yi,yi*) is the logarithmic loss [31] between the prediction scores yi and the true prediction scores yi*, T is the number of nodes in a tree, N is the number of training samples, γ, λ, and α are constants, and ‖.‖1 and ‖.‖2 are L1 and L2 norms, respectively.

The goal of training is to push the class probabilities *p*_m_ (Figure 5) as close as possible to the true classes in the training data. However, we also wanted to avoid overfitting to the training data. Therefore, our training objective should also prevent the model from becoming too complex. Accounting for these two objectives, the XGBoost optimization algorithm uses the cost function explained in Figure 6.

While the logistic loss term in Figure 6 (leftmost term) penalizes the discrepancy between the ensemble’s prediction and the ground truth, the rest of the terms prevent trees from overfitting by penalizing the number of nodes (*T*) as well as the magnitude of their prediction scores (*y*).

**Figure 7:**
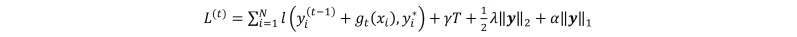
Cost function for training a new tree added at iteration t; where gt(.) is the prediction score provided by the new tree. See Figure 6 caption for more details on the parameters.

In the gradient boosting method, trees are added to the ensemble one by one. The ensemble starts with one tree, which is fit to the training data using the cost function in Figure 6. At each iteration, a new tree is added to the ensemble such that it fits to the residual error of the existing trees on the training data. Concisely, the new tree complements the existing trees such that, at iteration *t*, the cost function in Figure7 is minimized.

The parameters of the new tree are chosen such that *L*^(t^^)^ is minimized. In this way, the ensemble gradually fits to the training data. To find out when to stop adding new trees to the ensemble, we calculated the cross-validation error within the training dataset at each iteration. As the number of trees increase, this error decreases. However, after a certain point, the error starts to increase due to overfitting. We stopped adding new trees at that point, and evaluated the resulting classifier on the test set (see Classifier Evaluation).

#### Hyperparameter Tuning

We tuned the hyperparameters of the XGBoost classifier by grid search, and used data from 10% of participants. Within this subset of data, we performed a 10-fold cross-validation to estimate the area under the curve (AUC; see Classifier Evaluation). We chose the set of parameters on the grid that maximized this AUC.

The parameters included in hyperparameter tuning were *γ*, L1 regularization weight (*α*), L2 regularization weight (*λ*), learning rate, maximum tree depth, subsampling fraction (*r*), and feature subsampling fraction (*s*). Subsampling fraction, *r* ∈ (0,1), determines the fraction of training data samples that are seen by each tree during training, while features subsampling fraction, *s* ∈ (0,1), is the fraction of features that are seen by each tree node. After finding the optimal value of these hyperparameters, we trained and evaluated classifiers on the whole dataset.

#### Classifier Evaluation

Our goal was to create algorithms that could determine the semantic locations for unseen individuals, so we trained and evaluated the classifiers using a subject-wise cross-validation scheme. Specifically, we randomly selected 70% of the subjects to train the classifier, and used the remaining 30% to evaluate its prediction accuracy. We repeated this procedure 100 times. The distribution of prediction errors on held-out participants used as *test* provides an unbiased estimate of the prediction error of the algorithm for the population from which our dataset is sampled [32]. Therefore, we could tell how well our classifier would generalize to new, unseen individuals.

To calculate the prediction error in each round of cross-validation, we estimated the receiver operating characteristic curve, and calculated the AUC. The AUC ranges between 0 and 1, with 0.5 indicating chance level performance. The advantage of using AUC is that it is robust to the imbalance in the number of samples in the classes. Therefore, by iterating over all participants as *test*, we obtained a good estimate of the classifier’s accuracy.

### Relationship Between Semantic Location and Depression and Anxiety

We evaluated the relationship between the amount of time participants spent at each semantic location and their level of depressive and anxious symptoms, measured by PHQ-9 and GAD-7, respectively. We performed two analyses. First, we calculated Pearson’s correlation between the scores and the time spent in each location, across all participants. For the second analysis, we divided participants into depressed and nondepressed, as well as anxious and nonanxious, based on their scores. For depression, we defined the two groups by considering participants who consistently had PHQ-9 <10 (termed nondepressed) or PHQ-9 >10 (termed depressed) across all three assessment time points. Likewise, for anxiety, we defined the two groups by considering participants who consistently had GAD-7 <10 (termed nonanxious) or GAD-7 >10 (termed anxious). Therefore, in both analyses, we excluded the participants who crossed the PHQ-9=10 or GAD-7=10 thresholds. The main reason was that these participants could not be reliably classified. Furthermore, if we had included them, it would have added two additional categories (those who improved and those who got worse), which would have reduced power. It is also unclear how we would interpret any relationships with participants transitioning from one clinical state to another. After dividing subjects into these groups, we compared the duration of time that participants spent at each semantic location between the groups, using two-sample *t*-tests.

## Results

### Participant Statistics

A total of 208 individuals passed the eligibility criteria for participating in our study, and were recruited. One participant did not install the software on their phone, and another had invalid GPS data. These two participants were removed from all analyses. Of the remaining 206 participants, 22 (10.7%) stopped providing data before the end of the 6-week period. However, many continued to send data after the end of 6 weeks, with 27 (13.1%) providing more than 60 days of data.

The 206 participants included in the analyses were 170 females (82.5%) and 36 males (17.5%). Participants’ ages ranged between 18 and 66 years, with a mean of 39.3 (SD 10.3). The participants’ locations were diverse, covering most of the populated states and major cities in the United States. Most of these locations (86.8%, 178/206) were in “mostly urban” areas, as defined by the United States Census Bureau [33], while 12.1% (25/206) were in “mostly rural” areas. The rural or urban condition for the location of the remaining 3 participants could not be determined. The average depression score (PHQ-9) was 9.72 (SD 5.10), and the anxiety score (GAD-7) was 9.01 (SD 5.41). These values show that our participants had a wide distribution of depression and anxiety symptoms.

In response to a question on employment status, 61.2% (126/206) indicated that they were employed, 20.9% (43/206) were unemployed, 8.3% (17/206) had a disability which prevented them from working, and 1.9% (4/206) were retired. Sixteen participants (7.8%, 16/206) did not specify their employment status. Of the 126 employed participants, 98 (77.8%) had one, 23 (18.3%) had two, 4 (3.2%) had three, and one (0.8%) had four jobs. In addition, of these 126 participants, 36 (28.6%) worked in more than one location.

### Semantic Location Self-Reports

**Figure 8:**
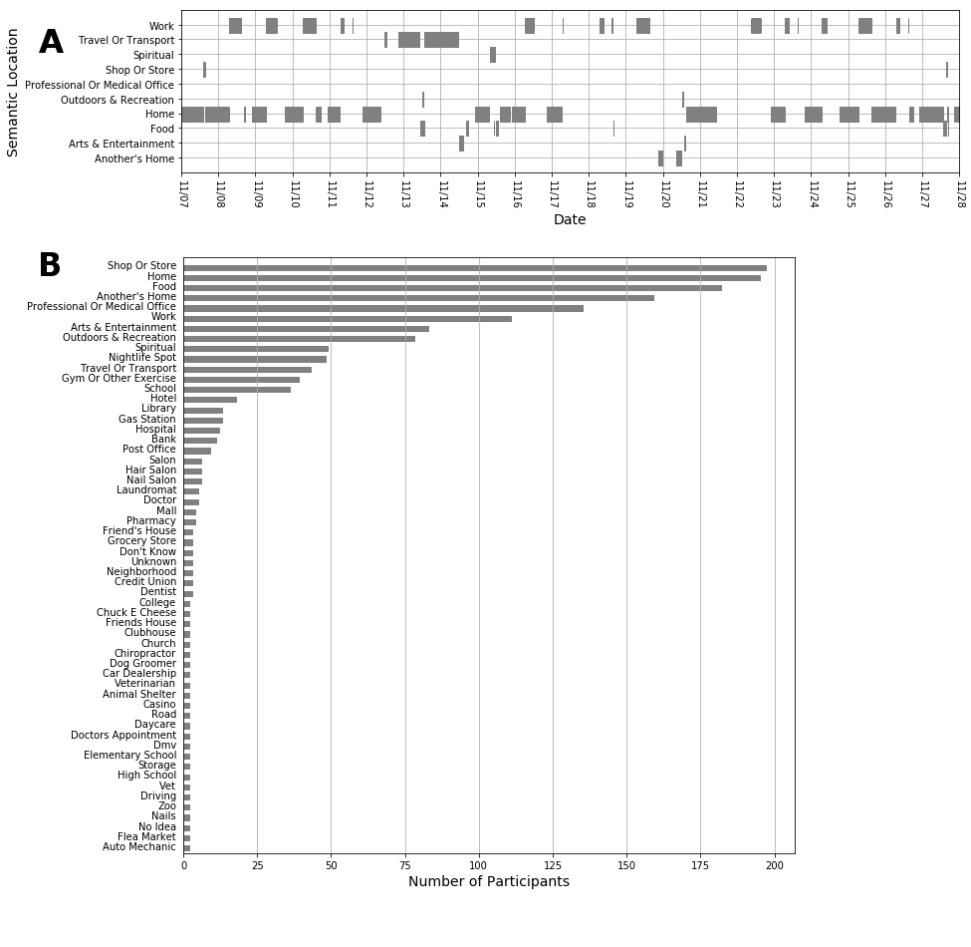
(A) Location report data from one example participant, collected between 11/07/2015 and 11/28/2015. Each rectangle shows the period of time the participant has been in a specific location. The sensor data during that time period is used to create a feature vector, which is then used to detect that semantic location. (B) Top locations visited by all participants, sorted by how many participants visited them. As the total number of unique reported locations was 370, we only included the ones that had been visited by at least two participants.

The semantic locations reported by the participants were diverse. While most participants reported the predefined locations in Purple Robot, as the example in Figure 8 A shows for one participant, many participants defined their own semantic locations by selecting “Other” and typing in their desired semantic location name. The total number of distinct location types reported by all participants was 370; however, only a small fraction of these locations was consistently reported by most participants (Figure 8 B). Therefore, apart from a few categories which need to be considered for future studies (eg, School and Library), most of the visited locations were among the locations that we had considered in the initial design of our mobile app.

### Classifier Hyperparameters

The optimized hyperparameters for the XGBoost classifier were the following: for sensor-only classification, we set the number of trees to 200, the fraction of samples seen by each tree to 0.2, and the fraction of features to 0.5. For classification based on both sensor and Foursquare features, these three parameters were set to 300, 0.25, and 0.2, respectively. In both scenarios, we set *γ*=0.4, *λ*=1, *α*=0, the maximum depth of decision trees to 4, and learning rate to 0.025. Given these parameter values, our training procedure was substantially regularized.

### Predicting Semantic Location

We first measured how accurately Foursquare could detect the semantic locations reported by participants. To obtain the locations detected by Foursquare, we used the GPS coordinates of that location, and queried Foursquare about its closest match to that location. We then compared the results to the locations reported by participants, and calculated the AUC for each category. The results are shown in the left column of Table 2. While Foursquare could detect Shop or Store with an average AUC 0.76, its AUC for Home was close to the chance level. Foursquare did not have location categories equivalent to Work, Another’s Home, or Spiritual, and therefore the AUCs for these categories could not be calculated. On average, the accuracy of Foursquare in detecting 8 semantic locations was approximately 0.62.

**Table 2:** Mean area under the curve (AUC) in detecting each location category, using Foursquare only, mobile phone sensor features only, and both. Note that we could not use Foursquare to detect Work, Another’s Home, or Spiritual locations; hence there are no results.

Semantic Location	Foursquare	Sensor	Sensor+Foursquare
Travel or Transport, mean (CI)	0.54 (0.49-0.60)	0.79 (0.72-0.86)	0.84 (0.78-0.91)
Nightlife Spot, mean (CI)	0.61 (0.53-0.72)	0.87 (0.78-0.94)	0.89 (0.79-0.95)
Spiritual, mean (CI)	N/A	0.82 (0.75-0.88)	0.87 (0.80-0.92)
Outdoors & Recreation, mean (CI)	0.59 (0.53-0.64)	0.81 (0.71-0.88)	0.86 (0.75-0.92)
Arts & Entertainment, mean (CI)	0.67 (0.61-0.73)	0.88 (0.85-0.91)	0.92 (0.88-0.95)
Work, mean (CI)	N/A	0.86 (0.82-0.90)	0.87 (0.83-0.91)
Professional or Medical Office, mean (CI)	0.65 (0.58-0.73)	0.85 (0.80-0.91)	0.88 (0.83-0.93)
Another's Home, mean (CI)	N/A	0.77 (0.69-0.82)	0.83 (0.75-0.89)
Food, mean (CI)	0.64 (0.59-0.68)	0.79 (0.74-0.83)	0.83 (0.78-0.87)
Home, mean (CI)	0.53 (0.51-0.56)	0.96 (0.95-0.97)	0.96 (0.95-0.97)
Shop or Store, mean (CI)	0.76 (0.73-0.79)	0.86 (0.82-0.90)	0.89 (0.85-0.92)
Mean AUC	0.62	0.84	0.88

We wanted to determine whether mobile phone sensors alone could detect the semantic location of participants. We used 45 features that were extracted from a variety of sensors during the time that the participant was visiting a location (see section: Sensor Features). We trained the XGBoost classifiers to map these features to semantic locations, and tested these classifiers on participants that they had not seen during training. Compared to Foursquare, the AUC of detecting certain locations was considerably higher (Table 2, middle column). Specifically, using the sensors instead of Foursquare yielded AUCs that were on average more than 20% greater (Table 2, middle column). This increase was mostly evident for Home, Nightlife Spot, and Travel or Transport categories. Overall, the average AUC for all semantic locations increased to 0.84. Therefore, not only could we use phone sensors alone to detect semantic locations, but their performance was considerably better than Foursquare.

Next, we used both Foursquare and phone sensor data to see if this approach could further increase the accuracy of our classifiers. To this end, we added two extra features to the 45 features that we previously used for training the classifiers: the Foursquare location type, which was represented by a binary vector with 9 elements (each corresponding to one category); and the distance to the nearest Foursquare location. Therefore, the total number of features increased to 55. Using this new feature set further increased the average AUC to 0.88 (Table 2, right column). This increase was mostly evident in detecting Food, Shop or Store, Art & Entertainment, and Spiritual categories. Therefore, augmenting mobile phone sensor features with Foursquare data made our classifiers better at detecting semantic locations.

**Figure 9:**
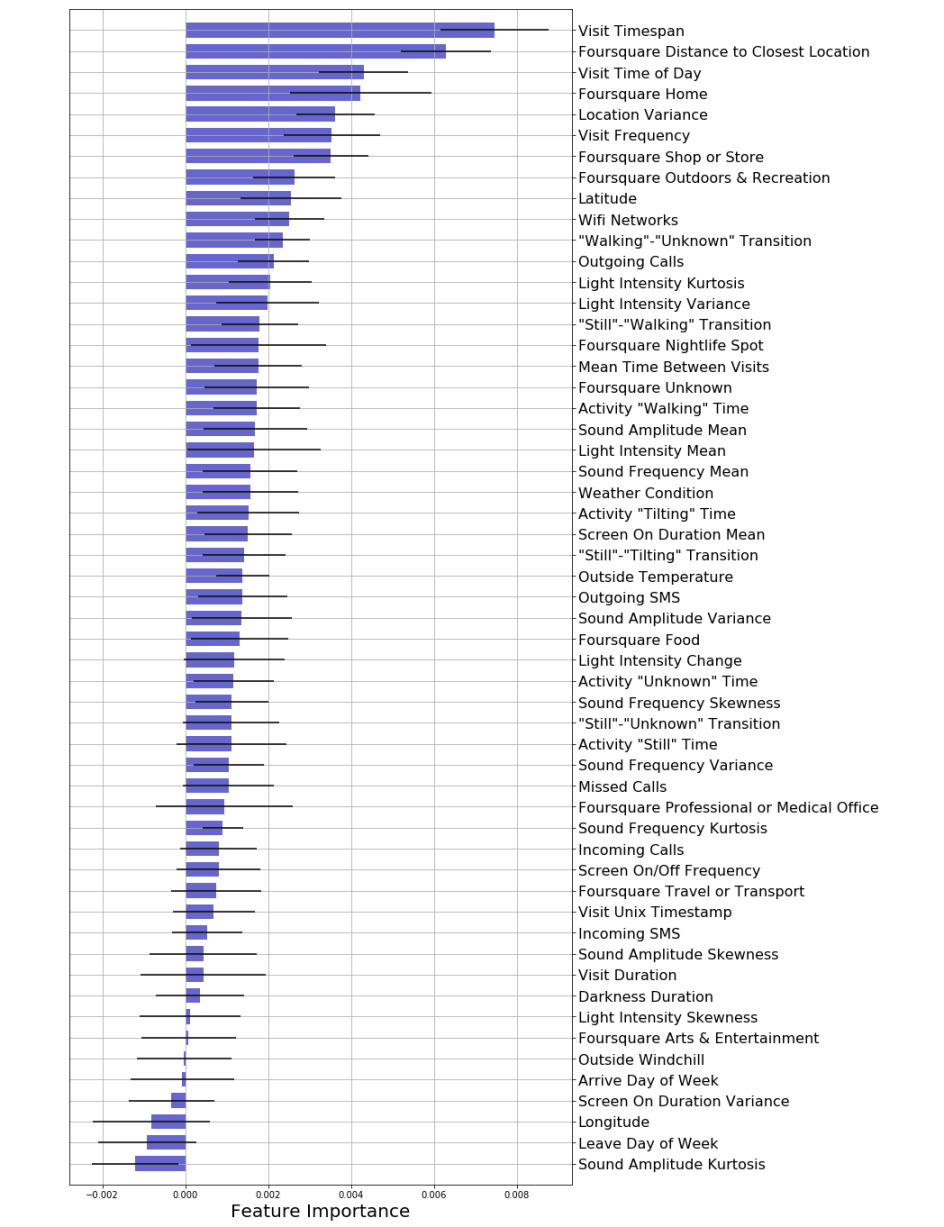
Mobile phone sensor feature importance in detecting semantic locations. Features are sorted based on their importance, from top to bottom. The importance of each feature is calculated by computing the decrease in the cross-validated area under the curve when that feature is removed from the feature set. Negative values indicate an increase in performance. Each value is the mean feature importance across cross-validation folds, and error bars show the standard error of the mean.

Finally, we asked which features contributed the most to detecting semantic locations by estimating their *importance*. To obtain feature importance for each feature, we removed that feature from the training data and calculated the resulting change in the cross-validated AUC. The results are shown in Figure 9, with features sorted by their importance. While features such as Visit Timespan, Location Variance, Latitude, Number of Wi-Fi Networks, Visit Duration, and Visit Frequency have the highest importance, several features have close to zero or negative importance, meaning that their removal does not affect (or even slightly improves) the performance of the classifiers. These features include some of the sensor features as well as Foursquare features. However, one should note that each of these effects are generated by removing only one feature from the feature set, and the collective effect of removing multiple features might be different. Nevertheless, it seems that most sensor and Foursquare features are useful in distinguishing semantic locations.

### Relationship Between Semantic Location and Depression and Anxiety

We evaluated the relationship between the time spent at different semantic locations and the level of depression and anxiety symptoms, measured by PHQ-9 and GAD-7, respectively. First, we evaluated the linear correlation between these two groups of variables (Table 3). When considering individual correlations, some were statistically significant (*P*<.05). Notably, the duration of time spent at Spiritual locations is negatively correlated with depression and anxiety scores, for 3 of 6 assessments. When we consider the total number of 66 comparisons between all semantic locations and depression and anxiety scores, we cannot rule out the possibility that these significant correlations are generated by chance. However, because these calculations are not independent, conservative corrections (such as a Bonferroni correction) may not be appropriate [34].

**Table 3:** Linear correlation coefficients (*r*) between time spent at semantic locations and depression (PHQ-9) and anxiety (GAD-7) scores. Values show the median of 1000 bootstrap estimates of *r*. Italicized values indicate coefficients that are significantly (*P*&lt;.05) different from zero. However, by correcting for multiple comparisons (66 comparisons here) we cannot rule out the possibility that these correlations are a result of chance.

	PHQ-9 Week 0	PHQ-9 Week 3	PHQ-9 Week 6	GAD-7 Week 0	GAD-7 Week 3	GAD-7 Week 6
*Home*	0.057	0.073	0.089	0.083	0.101	0.097
Shop or Store	-0.010	0.0183	-0.020	0.001	-0.030	-0.038
Work	-0.084	-0.139	-0.140	-0.083	*-0.176*	-0.085
Food	-0.088	-0.093	*-0.152*	-0.089	-0.086	-0.115
Another's Home	0.046	-0.065	-0.064	-0.016	-0.003	0.000
Professional or Medical Office	0.029	*0.096*	0.049	-0.069	0.019	0.051
Outdoors & Recreation	0.016	-0.123	-0.101	-0.065	-0.131	-0.109
Arts & Entertainment	*-0.172*	-0.092	-0.090	-0.044	-0.055	-0.057
Travel or Transport	-0.070	-0.037	*-0.113*	0.082	0.012	*-0.088*
Spiritual	-0.041	-0.078	*-0.147*	-0.094	*-0.143*	*-0.168*
Nightlife Spot	*-0.126*	*-0.173*	-0.045	0.041	-0.063	-0.045

We also performed a group difference analysis, by dividing the participants into two groups (once based on their depression scores, and another time based on their anxiety scores). We compared the duration of time participants spent at each semantic location between these groups. For depression, the nondepressed group consisted of 51 participants and the depressed group consisted of 68 participants. The remaining 88 participants crossed the PHQ-9=10 threshold between the assessments, and were excluded from this analysis because they could not be clearly classified. For anxiety, the nonanxious group consisted of 51 participants while the anxious group consisted of 61 individuals. The remaining 96 participants crossed the GAD-7=10 threshold and were excluded.

**Figure 10:**
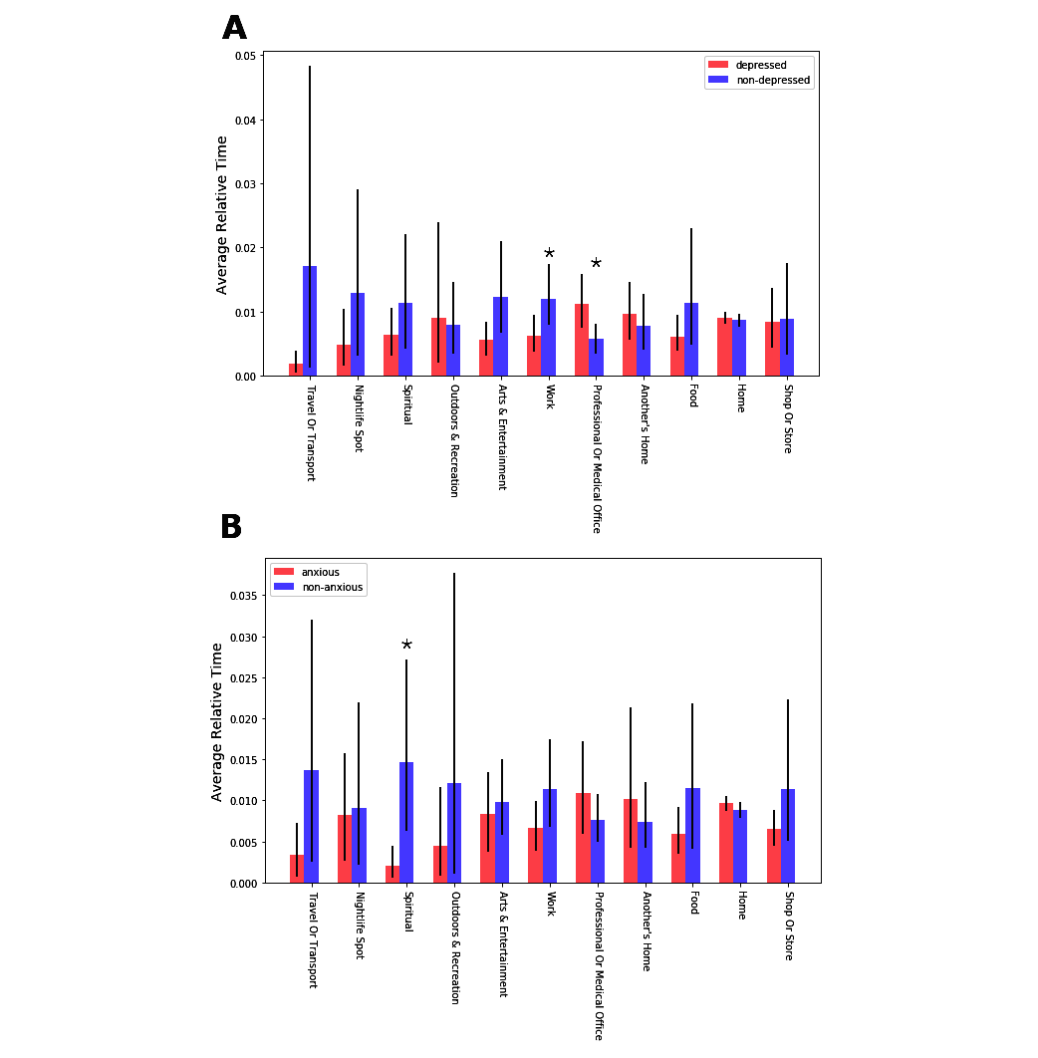
The relationship between semantic location visit duration, depression, and anxiety. Each bar shows the average time spent at each location by (A) depressed versus nondepressed and (B) anxious versus non-anxious groups, relative to the total time spent by all participants in that location. Error bars show 95% CIs. Both mean and CIs are obtained by bootstrapping over 1000 iterations. Stars indicate significant difference between the means, obtained using a 2-sample t-test at the P<.05 level. However, adjusting for multiple comparisons, these differences are all nonsignificant.

The results for depression are shown in Figure 10A. While the depressed and nondepressed groups seemed to have different distributions of time spent across locations, these differences were significant (*P*<.05) only for two locations: the nondepressed group spent significantly more time at Work, while the depressed group had more time spent at a Professional or Medical Office. For the anxious versus nonanxious comparison (Figure 10B), the difference was only significant for the Spiritual category, with the nonanxious group spending more time in this location category, on average. Therefore, it seems that time spent at semantic locations contains some information about depression and anxiety, but these findings are not consistent.

## Discussion

### Principal Results

In this paper, we were able to detect the type of locations that individuals visited, using data passively collected from their mobile phones. The phone sensor data were especially crucial in detecting these semantic locations. Sensor features alone produced accuracies that were more than 20% greater than those reported by Foursquare, and combining the sensor features with Foursquare produced even greater accuracy. This result is not surprising since detecting semantic location based on GPS alone is not necessarily accurate, especially in urban areas [35], and can lead to detecting nearby locations instead of the actual location. Sensors, which are available on most mobile phones, can provide valuable information about the type of locations phone users visit, and can significantly improve the accuracy of these services.

The performance of the classifiers considerably varied across the location types. While Home could be detected with an AUC of above 0.95, the classification AUC for Another’s Home and Food was 0.83. This variability may have multiple causes. First, visits to certain locations, such as home or work, are more regular in time, which makes them easier to detect based on the time of visit. Another cause might be that some semantic locations such as Travel or Transport were less represented in the data, since participants visited those locations less often. This factor has likely made it difficult for classifiers to find the feature patterns that are distinct indicators of those locations. Finally, while some locations (eh, Home) have a clear definition, participants may have been confused about which location type to report for some other locations. For example, a participant might have had food in a store, and have reported that location as either “Food” or “Shop or Store”. Overall, although classification performance varied across different semantic locations, it significantly benefited from incorporating mobile phone sensor data.

While we could detect the types of locations, we found only few significant relationships between the amount of time spent in those locations and self-reported symptoms of depression and anxiety. Furthermore, these few relationships were weak and inconsistent. This failure may have multiple explanations. First, our categorization of semantic locations was based largely on Foursquare categories, which was not developed with mental health or wellness in mind and may not be accurate, useful, or relevant to mental health. These categories were also often imprecise (eg, “Professional or Medical Office”). For mental health research, we may need to create location categories that are mostly relevant to the factors that influence mental health.

Second, the lack of a consistent relationship between semantic location and depression or anxiety may reflect larger problems in the literature. Past research has examined smaller, discrete samples of participants, such as university students [2,4,8,36] or residents of the same city [3,37]. This study sample was geographically diverse, with a broader sample of the American population. This diversity in location enriched our dataset by including people from rural and urban areas, and different climates, cultures, and lifestyles. While this diversity helped us to obtain a better estimate of the accuracy of location detection in real-world applications, it may also reflect problems with increasing dimensionality, as this area of research moves towards more generalizable samples.

It is possible that this finding is accurate: that the kinds of places we go is *not* related to our level of depression or anxiety. This theory would suggest that the relationship between movement through geographic space and depression or anxiety [2-4,37] may be related to some other aspect of mobility patterns. For example, it may be that depression or anxiety is more related to the processes of getting to various locations, such as physical activity [10,38,39], than the actual locations themselves. Furthermore, low motivation in depressed individuals may decrease the likelihood of moving from a commonly visited location (such as home or work) and a less frequently visited place (such as a store or movie theater), but may have very little to do with moving from a less frequently visited place to home or work [40].

### Limitations

There are a number of limitations that need to be mentioned. First, when detecting semantic locations we did not consider the transitions between locations. Knowing the transition probabilities can be useful; for example, it may be more likely to visit Home after Shop or Store. One reason for not considering transitions was that we only considered the top 11 most-visited locations for the classification problem, and therefore the sequence of semantic locations in the training data were not necessarily consecutive in time. Another reason was the existence of gaps in the data, which caused further separation between consecutive visits. Incorporating transition probabilities in detecting semantic locations, when possible, will likely increase the classification accuracy of the resulting algorithms.

Second, semantic locations may have signatures that we failed to capture through our phone sensors. For example, the type of phone apps people use, or individuals who they contact, can be a good distinguishing feature between locations. Using such sources of information as features in future studies may improve the performance of semantic location detection.

Third, our study participants differed from the general population in a few aspects. Approximately 83% of the participants were women, significantly different from 50.8% in the general population of the United States [41]. Furthermore, nearly 21% of the participants were unemployed, compared to the nationwide estimate of 5% unemployment [42]. Finally, we only included individuals who owned smartphones, while approximately 28% of Americans do not own such phones [43]. In addition, our inclusion of people with only Android phones excluded 41% of smartphone users who use phones with other operating systems [44]. Census data shows that owing a smartphone is associated with certain demographic variables such as age, education, and income [43]. Therefore, our inclusion criteria might have affected the study sample.

Fourth, the assessment of depression and anxiety in this study was based on self-report, and therefore may not generalize to assessments based on diagnostic interview. A clinical diagnosis usually involves an in-depth interview and consideration of confounding factors, based on the criteria in the Diagnostic and Statistical Manual of Mental Disorders [45,46]. In our study, the assessment was solely based on Web-based self-reported PHQ-9 and GAD-7 scores, and therefore our study sample may be different from a clinical sample. It is likely that we would find a stronger relationship between mental health state and the type of visited locations in a clinical sample, compared to what we found in this study. Nevertheless, electronic assessment of depression has been used and validated by many previous studies [47,48].

Fifth, data collection took place from late October to early February, and thus most participants were providing data during the winter holiday season. While the geographic diversity of the sample allows us to account for variations in weather (eg, participants from Florida experienced a much different climate than those in Minnesota), we recognize that holiday-related travel, such as spending time at other family members’ homes, and holiday-related time away from work presents a departure from an individual’s typical behavior. The holiday season may have served as a confounder, as participants may have been engaged in activities not representative of how they would behave during other times of the year. Furthermore, the 6-week study period may not have been long enough to detect changes or meaningful relationships between behavioral patterns and mood. Ultimately, we aim to develop models to ascertain the relative components of these factors. However, as this is a relatively new field of inquiry, the timing and length of this study protocol may have interfered with our ability to detect true signals.

### Conclusions

In conclusion, mobile phone sensors promise considerably more accurate estimations of individuals’ daily life behaviors. In this study, we have shown that semantic location (the type of locations that people visit) can be detected using a combination of phone sensors and a mapping service such as Foursquare. We performed this study in a sample that was diverse in terms of geographic location, climate, education, employment, and lifestyle. However, there were no consistent relationships between the time spent at different locations and depression or anxiety. Future research should focus on those semantic locations that are more likely to be relevant to depression or anxiety. In addition, longer studies that extend across seasons, and larger studies that are more adequately powered to manage the level of dimensionality in human subject data, will be better positioned to investigate the relationships between semantic locations and mental health. The advancement of mobile phone technology will facilitate the design of these future studies.

## Abbreviations

API: application programming interface
AUC: area under the curve
EMA: ecological momentary assessment
FPG: Focus Pointe Global
GAD-7: Generalized Anxiety Disorder, 7-item
GPS: global positioning system
ID: identification
JITAI: just-in-time adaptive intervention
JSON: JavaScript object notation
PHQ-9: Patient Health Questionnaire, 9-item
SD: standard deviation
SMS: short message service
XGBoost: extreme gradient boost
